# ATP Metabolism of Astrocytes: Consumption, Regeneration and Restoration

**DOI:** 10.1007/s11064-025-04604-7

**Published:** 2025-11-12

**Authors:** Ralf Dringen, Gabriele Karger, Ulrike Winkler, Johannes Hirrlinger

**Affiliations:** 1 Center for Biomolecular Interactions Bremen, Faculty 2 (Biology/Chemistry), University of Bremen, PO. Box 330440, D-28334 Bremen, Germany; 2 Center for Environmental Research and Sustainable Technologies, Leobener Strasse, D-28359 Bremen, Germany; 3 Carl-Ludwig-Institute for Physiology, University of Leipzig, Liebigstrasse 27, D-04103 Leipzig, Germany; 4 Department of Neurogenetics, Max-Planck-Institute for Multidisciplinary Sciences, Hermann-Rein-Str. 3, D-37075 Göttingen, Germany

**Keywords:** Astrocytes, ATP, Energy metabolism, Glycolysis, Oxidative phosphorylation

## Abstract

Astrocytes are essential partners of neurons and have many important functions in the brain. Almost all of these astrocytic functions require energy that is provided by cellular adenosine triphosphate (ATP). Accordingly, astrocytes contain a millimolar concentration of cellular ATP that is maintained by continuous and rapid regeneration from adenosine diphosphate (ADP) and adenosine monophosphate (AMP), the main products of cellular energy-consuming reactions. In this article we describe the current knowledge on the cellular content, the consumption and the metabolic regeneration of ATP in astrocytes, explore the consequences of an application of metabolic inhibitors on astrocytic ATP metabolism and summarize the importance of endogenous energy stores and exogenous energy substrates for the maintenance of a high cellular ATP content. In addition, we give insight in recent studies on the visualization of ATP in astrocytes by genetically encoded ATP sensors, summarize the importance of astrocytic ATP release and extracellular ATP processing and discuss recent data on the restoration of ATP in ATP-deprived astrocytes. The current knowledge on the ATP metabolism of astrocytes clearly demonstrates the high potential of this important brain cell type to flexibly use different metabolic pathways and a broad range of endogenous and exogenous sources to maintain, regenerate and restore cellular ATP levels. These processes secure that ATP is continuously available for the many ATP consuming processes that enable astrocytes to perform their functions in the healthy brain.

## Introduction

Adenosine triphosphate (ATP) is the main energy currency of living cells [1, 2]. Every text book of biochemistry describes in detail the structure of ATP, the energy-rich phosphoric acid anhydride bonds that provide the energy needed for most energy-dependent cellular reactions, as well as the main pathways that are involved in the regeneration of ATP from adenosine diphosphate (ADP). Research on ATP consuming and regenerating pathways has been a fundamental part of biochemical research in the past [1], but it recently regained substantial interest in neuroscience due to the growing evidence that alterations of brain energy metabolism play an important role in neurological and neurodegenerative diseases [3–7].

Concerning ATP metabolism, the brain is a particular prominent organ as it needs 20% of the total energy consumed by the human body and metabolises 20% of glucose and oxygen. Most of this oxygen has been discussed to serve as electron acceptor for oxidative phosphorylation in neurons to fuel membrane dependent processes as well as for the information transfer at synapses [8], although this view has recently been challenged and the energetic costs of astrocytic functions has been revisited [9]. Considering the high overall consumption of glucose and oxygen by the brain, also glial cells contribute substantially to the catabolism of glucose in brain to regenerate the ATP that is needed to fuel glial functions. Astrocytes represent the majority of non-neuronal cells in the central nervous system [10] and account for 20% to 40% of all brain cells [11]. Astrocytes have a plethora of important functions in brain as partners of neurons [12, 13]. For example, astrocytes have pivotal roles in brain energy metabolism [14–17], in the regulation of the extracellular milieu [13, 18], in the defence against reactive oxygen species and toxins [19–21], in neurotransmitter metabolism and neurotransmission [22–25] as well as in cognitive processes and memory formation [26–29]. Concerning ATP consumption, especially the active buffering of extracellular K^+^ concentrations is energetically costly as it involves the ATP-consuming Na^+^-K^+^-ATPase [30]. Taking this active buffering of K^+^ into account, it has been estimated that the energetic costs of astrocyte functions are similar to that of neurons [9], underlining the crucial importance of astrocytic ATP metabolism for the brain.

**Fig. 1:**
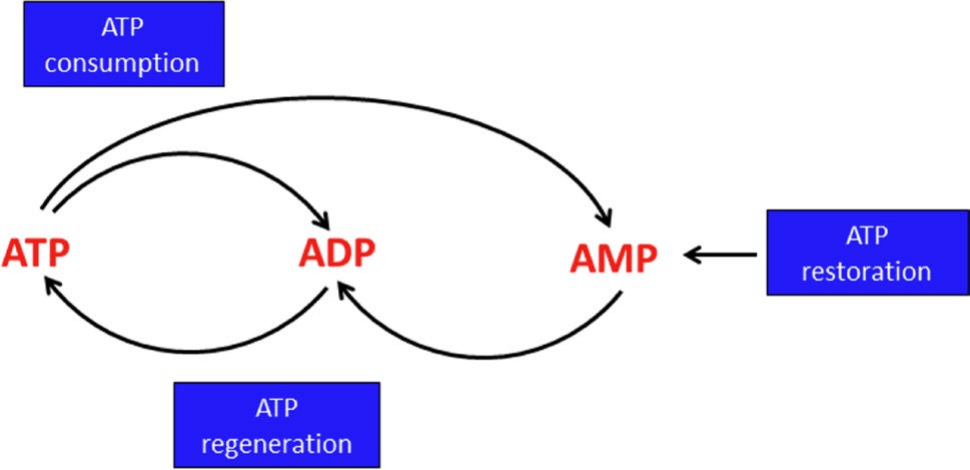
ATP consumption, regeneration and restoration in astrocytes. Most ATP consuming reactions generate ADP or AMP and these nucleoside phosphates are used as substrates for ATP regeneration. AMP is phosphorylated by adenylate kinase to ADP which is subsequently used as substrate of ATP regeneration by glycolytic substrate level phosphorylation or mitochondrial oxidative phosphorylation. After depletion of the total cellular pool of adenosine phosphates, ATP is restored via net synthesis of AMP from appropriate precursor substrates and its subsequent phosphorylation to ATP

Many reactions that help astrocytes to fulfill their functions in the brain require ATP as substrate (Table 1). The cellular ATP metabolism can be separated into three main aspects: (1) the consumption of ATP by various cellular enzymatic reactions and processes, (2) the continuous regeneration of ATP from ADP and adenosine monophosphate (AMP) and (3) the restoration of the normal high level of adenosine phosphates in ATP-depleted cells by synthesis of new AMP that serves as building block for subsequent phosphorylation to ATP (Fig. 1).

**Table 1:** Selected ATP consuming reactions in astrocytes

Type of reaction	Processes	Selected examples	References
Pathways and reactions that produce ADP from ATP			
	Transport pumps	Na^+^-K^+^-ATPase	[31]
		H^+^-ATPase (lysosomes)	[32]
		ABC transporters	[33]
		Ca^2+^-ATPases	[34]
	Phosphorylation of metabolites	Hexokinase	[35]
		Phosphofructokinase	[19]
		Creatine kinase	[36]
		Pyruvat carboxylase	[37]
		Adenosine kinase	[38]
	Phosphorylation of proteins by protein kinases	mTOR signaling pathway	[39]
		PI3/proteine kinase B signaling pathway	[40]
		Jak/STAT signaling pathway	[41]
		AMP-activated kinase	[42]
	Synthesis of small molecules	Glutamine synthetase	[43]
		Glutathione synthesis	[20, 21]
Processes and enzymes that produce AMP from ATP			
	Amino acid activation for protein synthesis	Amino acyl tRNA synthetases	[44]
	Activation of fatty acids for β-oxidation	Long-chain acyl-CoA synthetases	[45]
	Formation of cAMP	Adenylyl cyclases	[46]
Processes and enzymes that consume the adenosine moiety of ATP			
	Export of ATP from the cells	vesicular ATP release	[47]
	Synthesis of RNA	RNA polymerase II	[48]
	Synthesis of S-adenosylmethionine	Methionine adenosyl transferase	[49]
	Synthesis of nicotinamide-and flavine-containing coenyzmes	NAD^+^ synthesis	[50]
	Formation of Coenyzme A	CoA synthase	[51]

**Fig. 2:**
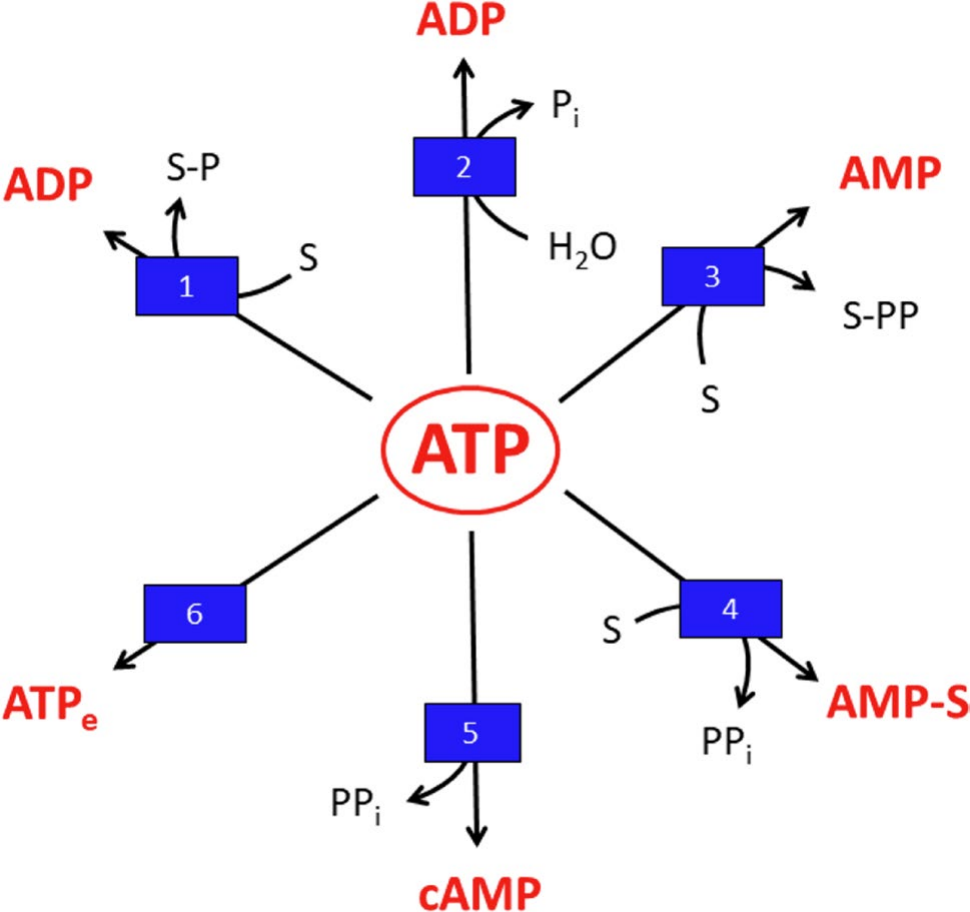
ATP consuming reactions in astrocytes. (1) Transfer of a phosphate group from ATP onto a substrate (S) to generate the phosphorylated substrate (S-P). (2) Hydrolysis of ATP to ADP and inorganic phosphate (P_i_). (3) Transfer of a pyrophosphate group (PP) to a substrate (S) to generate a pyrophosphorylated intermediate or product (S-PP). (4) Transfer of the AMP moiety of ATP to an acceptor substrate to form an adenylated intermediate and inorganic pyrophosphate (PP_i_). (5) Formation of cyclic AMP (cAMP) by adenylyl cyclases. (6) Release of cellular ATP from the cells to provide extracellular ATP (ATP_e_)

The main astrocytic reactions that consume ATP are shown in Fig. 2. The enzymes using ATP as substrate in astrocytes belong to a broad range of enzyme classes (EC), including EC2 (transferases i.e., hexokinase), EC3 (hydrolases i.e., NTPase, nucleoside triphosphatases), EC4 (lyases i.e., adenylyl cyclases), EC5 (isomerases i.e., dynein ATPase), EC6 (ligases i.e., glutamine synthetase) and EC7 (translocases i.e., Na^+^-K^+^-ATPase).

In many ATP-consuming reactions, the energy conserved in the distal anhydride bond is used and ADP is generated as product (Fig. 2; Table 1). Such reactions are involved in the phosphorylation of metabolites (Table 1). In addition, a large number of protein kinases use ATP to phosphorylate their protein targets, thereby regulating signaling and metabolism [39–42]. Also, the establishment of ion gradients by ATP-dependent active transport processes, for example by the Na^+^-K^+^-ATPase, involves the transient phosphorylation of the transport protein (Table 1). Moreover, the synthesis of important small biomolecules by astrocytes consumes ATP and produces ADP, such as the synthesis of glutamine and glutathione (Table 1).

For the regeneration of cellular ATP from ADP astrocytes use the pathways that are well known for most eukaryotic cells [52, 53], glycolytic substrate level phosphorylation and mitochondrial oxidative phosphorylation [14, 54]. In addition, accumulating ADP can be rapidly phosphorylated by creatine kinase (CrK) to ATP on the expense of the creatine phosphate (CrP) that is present in substantial amounts in astrocytes and serves as emergency buffer of high energy phosphates [55].

An additional group of ATP consuming enzymes produces AMP and pyrophosphate (Fig. 2). Such reactions include the activation of amino acids and fatty acids via adenylated intermediates to amino acyl-tRNAs and acyl-CoA thioesters (Table 1) that are used as substrates for proteins synthesis and fatty acid metabolism, respectively. Pyrophosphate is also generated during the formation of cyclic AMP (cAMP) by adenylyl cyclases (Table 1). The pyrophosphate generated is hydrolysed by phosphodiesterases [46, 56], while AMP is phosphorylated by adenylate kinase [57, 58] to ADP that is subsequently phosphorylated to ATP.

ATP is also consumed in astrocytes by ATP export [59] as well as by the use of the adenylate moiety of ATP as building block (Fig. 2) to synthesize AMP-containing cofactors, such as nicotinamide adenine dinucleotide (NAD^+^) [50] or nucleic acids. In addition, during severe impairment of ATP regeneration, the total pool of adenosine phosphates (ATP plus ADP plus AMP) is lowered in astrocytes, most likely by further metabolism of the accumulating AMP [55, 60]. To compensate for such losses in the total cellular pool of adenosine phosphates, the synthesis of new AMP is required (Fig. 1) to enable astrocytes to restore their normal high cellular ATP content.

Despite of a broad range of astrocytic functions that continuously consume ATP (Table 1), the ATP content of astrocytes appears to be rather stable and the cellular contents of ADP and AMP are very low, demonstrating that ATP is efficiently regenerated from ADP and AMP. In this article, we summarize the current knowledge on the various facets of ATP metabolism of astrocytes, including ATP consumption, regeneration and restoration (Fig. 1). We will cover the consumption of ATP in astrocytes by various cellular reactions as well as by ATP release and will discuss the regeneration of cellular ATP from ADP and AMP. In this context we will also explore the ability of astrocytes to use endogenous and exogenous substrates as fuels to provide the energy needed for ATP regeneration. Moreover, we will address the pathways involved in the synthesis of new AMP that is needed as building block for ATP regeneration in ATP-deprived astrocytes. Studies on the ATP metabolism of astrocytes have mainly been performed on astrocyte cultures derived from rat or mouse brain, for technical reasons. However, the now available genetically encoded ATP sensors as well as human astrocytes derived from inducible pluripotent stem cells (iPSC) provide new avenues to explore the ATP metabolism of astrocytes in the living brain and in human cells. Such rather new aspects and future perspectives for research on the ATP metabolism of astrocytes will also be addressed.

## ATP Content of Astrocytes

### Biochemical Quantification of the Total Cellular ATP Content of Astrocyte Cultures

ATP contents have frequently been quantified for astrocyte cultures derived from mouse or rat brain. Initially, ATP contents were mainly determined in acidic extracts of such cultures by enzymatic assays or high-pressure liquid chromatography (HPLC) (Table 2). These methods quantify the total cellular ATP content that was normalized to the total protein content of the respective cultures to obtain the specific ATP content. Most studies on cultured astrocytes determined specific ATP contents in the range between 20 and 40 nmol/mg (Table 2). Only few articles report substantially lower specific ATP levels of below 10 or even below 1 nmol/mg (Table 2). Potential reasons for the variations in reported specific ATP contents include different protocols for cell culturing, different methods to generate cell samples and different assay conditions. For example, components of the culture medium such as the absence or the presence of serum [55, 61] have been shown to affect the cellular ATP content, but such reasons are unlikely to explain the very low specific ATP contents reported by some groups (Table 2). However, it should be mentioned that substantial variations in the specific ATP contents can also be found for few individual astrocyte cultures that have been prepared and maintained by an identical protocol. In our hands, the specific ATP content of most of the rat astrocyte-rich cultures prepared and used for experiments was in the range between 25 and 35 nmol/mg, but a few individual cultures contained specific ATP levels as low as 15 nmol/mg [62] or as high as 45 nmol/mg [55]. At least the age of the cultures appears not to be an important parameter that significantly affects the specific ATP content of cultured astrocytes [55, 62].

Assuming that most ATP in cultured astrocytes is localized in the cytosol, the cytosolic ATP concentration can be calculated by making use of the specific ATP content of a culture and the specific cytosolic volume of 4.1 µL/mg [63]. A specific ATP content of 30 nmol/mg would thereby correspond to a cytosolic concentration of 7.3 mM. Accordingly, cellular ATP concentrations of cultured astrocytes were calculated to be in the range of 3 to 10 mM [62, 64, 65].

**Table 2:** Specific ATP contents of murine astrocyte cultures

Species	Specific ATP content (nmol/mg)	Cell extracts generated in	Assay	Reference
Rat	36.5 ± 2.5	PCA	HPLC	[66]
Rat	36.0 ± 6.4	PCA	Enzymatic luminometry	[55]
Rat	35.2 ± 3.4	PCA	Enzymatic luminometry	[67]
Rat	35.1 ± 1.8	KOH	HPLC	[68]
Rat	32.2 ± 4.7	PCA	Enzymatic luminometry	[60]
Rat	31.8 ± 6.6	PCA	HPLC	[69]
Rat	30.0 ± 5.0	PCA	Enzymatic luminometry	[70]
Rat	27.9 ± 4.7	PCA	Enzymatic luminometry	[62]
Rat	25.7 ± 0.7	TCA	Enzymatic luminometry	[71]
Rat	25.4 ± 2.9	PCA	HPLC	[72]
Rat	25.2 ± 0.6	PCA	Enzymatic-fluorometry	[73]
Rat	13.3 ± 0.8	PCA	Enzymatic luminometry	[61]
Rat	Around 6.5	TCA	Enzymatic luminometry	[74]
Rat	Around 6.5	TCA	Enzymatic luminometry	[75]
Rat	3.8 ± 0.4	Triton X-100	Enzymatic luminometry	[76]
Rat	Below 1	PCA	Enzymatic luminometry	[77]
Mouse	47.5 ± 1.7	PCA	HPLC	[78]
Mouse	47.3 ± 2.1	PCA	Enzymatic-fluorometry	[79]
Mouse	46.8 ± 3.5	PCA	Enzymatic-fluorometry	[80]
Mouse	39.7 ± 5.8	TCA	Enzymatic luminometry	[81]
Mouse	20.1 ± 2.5	PCA	HPLC	[82]

*HPLC* High pressure liquid chromatography,* PCA* Perchloric acid,* TCA* Trichloroacetic acid.

Main products of ATP consuming reactions are ADP and AMP (Fig. 2; Table 1). In untreated astrocytes the contents of these two nucleoside phosphates are rather low [66, 69, 72, 82, 83], representing around 10% (ADP) and 5% (AMP) of the total pool of adenylate phosphates [55, 60]. Accordingly, cultured astrocytes have a high adenylate energy charge (AEC: ([ATP] + 0.5 [ADP])/([ATP] + [ADP]+ [AMP])) [84] of around 0.9 [55, 60, 69, 72, 82].

In comparison to the high specific ATP content, the cellular contents of the other nucleoside triphosphates are much lower in cultured astrocytes [66, 69], consistent with the cellular need of ATP as general energy currency for a broad range of cellular reactions (Table 1), while other nucleoside triphosphates are used in a more limited number of specific processes.

### Monitoring of Cytosolic ATP Concentrations in Astrocytes by ATP Sensors

**Fig. 3:**
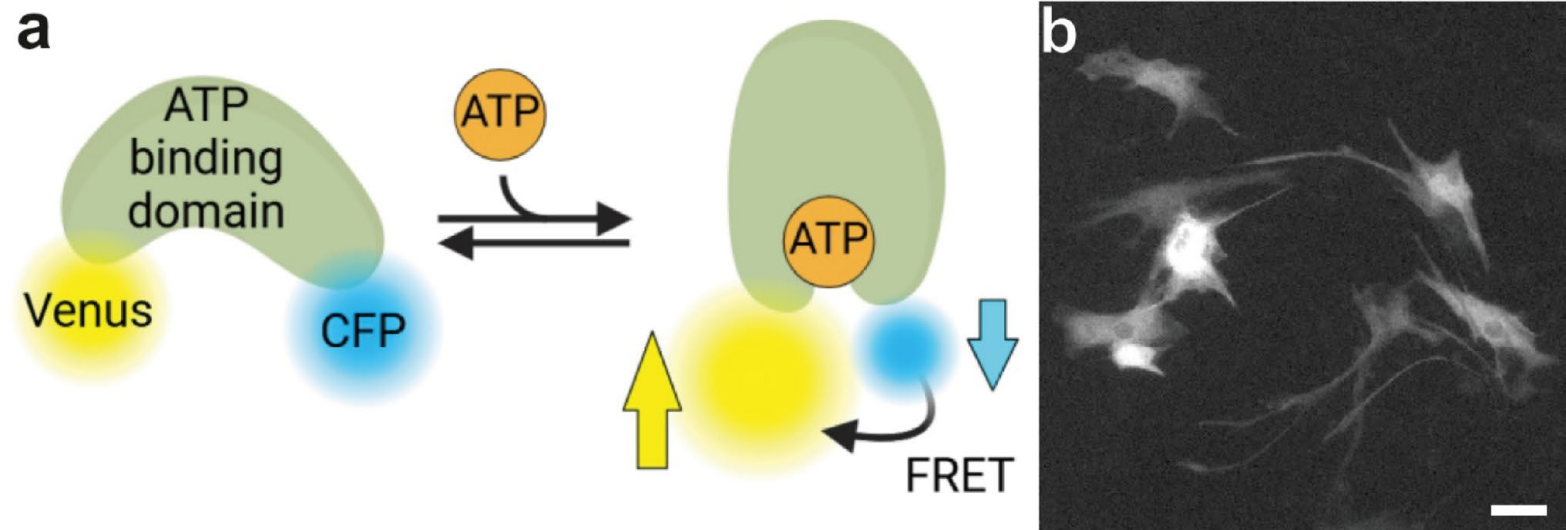
Imaging of ATP sensors to study ATP in astrocytes. **a** Principle of ATP sensors exemplified for the ATeam sensors [89]. Binding of ATP to the ATP-binding domain induces a conformational change resulting in a FRET-effect. Therefore, the fluorescence of the FRET-donor CFP decreases, while the fluorescence of the FRET acceptor Venus increases. **b** Primary cultured mouse astrocytes were transfected with the ATP sensor ATeam1.03YEMK to visualize cytosolic ATP. The Venus-channel of the sensor’s fluorescence is shown (scale bar: 50 μm). Panel a was created in BioRender. Hirrlinger, J. (2025) https://BioRender.com/1faqufa

The advent of genetically encoded, fluorescent sensors for metabolites (genetically encoded metabolite indicators; GEMI) has provided a novel and powerful approach to analyzing metabolites in cells with high spatial and temporal resolution. Such sensors have been developed for several metabolites and change their fluorescent signal depending on the concentration of their target metabolite (Fig. 3; for recent reviews see [85–88]). Specifically for ATP, a number of different GEMIs have been developed: the ATeam family [89] including the green-orange version GO-ATeam [90], QUEEN [91], MaLion [92], ChemoG-ATP_SiR_ [93], iATPSnFR1.0 [94] and iATPSnFR2 [95]. Furthermore, the Perceval and the PercevalHR sensors report the ATP/ADP ratio [96, 97]. As these sensors cover a rather broad spectrum of dissociation constants (k_D_), they are sensitive for different ranges of ATP concentrations. However, only few of these GEMIs have been used to study ATP dynamics in astrocytes. Also, sensors for monitoring extracellular ATP have been developed, like e.g. a modified ATeam sensor [98], the GRAB_ATP1.0_ sensor [99] and others (for a review see [59]).

The main advantages of these sensors include (1) cell-type specific expression allowing to analyze metabolites in a cell-type specific manner even in their natural environment both in situ and in vivo; (2) high spatial resolution which is only limited by the resolution of the microscope, allowing analysis of differences between single cells of the same cell type as well as of subcellular metabolite dynamics; (3) high temporal resolution allowing to record fast changes in the metabolite concentration, which – in combination with appropriate protocols like the inhibitor-stop approach [100] – also enable recording metabolic fluxes.

Major challenges using GEMIs are confounding factors like changes in pH which potentially also affect the fluorescence signal as well as the calibration of GEMIs to the actual concentration of the metabolite [101]. Such a calibration is especially difficult for ATP as it requires access of the metabolite to the intracellular space to precisely control its concentration within the cytosol as well as the inhibition of both cellular production and consumption of the metabolite. In the case of ATP, access of extracellular ATP to the cytosol requires cell permeabilization as no cellular transporters are available to equilibrate ATP in the extracellular and intracellular space. Furthermore, while cellular production of ATP can largely be inhibited by blocking glycolysis and mitochondrial oxidative phosphorylation, blocking all of the large number of ATP consuming processes in cells is impossible (for a detailed discussion see [64]). Consequently, many studies report changes of the fluorescence signal of the ATP sensors as proxy for the ATP-concentration, and only few reports provide data on actual, molar concentrations of ATP derived from ATP sensor imaging [64, 102, 103]. As an alternative approach, the “dual nanosensor” method has been developed, which takes advantage of two GEMIs which both report the same metabolite but with different dissociation constants k_D_ [64]. For cells expressing the different GEMIs a given change of the cytosolic concentration of ATP ([ATP]_c_) induced by a treatment will result in different changes of the GEMI signals due to the different k_D_-values of the GEMIs for ATP. The exact values of this pair of signal changes unequivocally depends on the basal [ATP]_c_, thereby allowing to calculate basal [ATP]_c_ and, consecutively, changes in [ATP]_c_ during treatment without permeabilization of cells [64].

In contrast to biochemical measurements of ATP in cell extracts which detect all the ATP present, sensor-based approaches allow to estimate the cytosolic concentration (Table 3) of unbound ATP (or in other cell compartments, if the sensor is genetically targeted to the specific organelle). Due to the difficulties of calibration, only few studies reported molar values of [ATP]_c_. Using a luminescent sensor in cultured cerebellar and hypothalamic astrocytes, which was calibrated by cell permeabilization by digitonin, a [ATP]_c_ of 1.5 mM and 1.4 mM was calculated [102]. Consistently, using the dual nanosensor approach, the [ATP]_c_ in cultured cortical astrocytes was estimated as 1.5 mM, while the concentration in astrocytes in acute cortical slices was calculated to be in the range between 0.7 mM and 1.3 mM [64]. As expected, these numbers (Table 3) are substantially lower than concentrations obtained from biochemical measurements in extracts that range between 3 and 10 mM [61, 62, 65, 81]. The quantification of ATP in acidic extracts of cultured astrocytes determines the total ATP content of the cultured cells, including ATP that is present in cellular organelles including the nucleus, mitochondria, and vesicles as well as ATP that is bound to proteins. These ATP sources are not accessible for detection by a cytosolic ATP sensor. Therefore, it is reasonable to assume that the basal free [ATP]_c_ of astrocytes is around 1 mM to 2 mM. As the K_M_ values for ATP consuming enzymes (for example hexokinase and creatine kinase) and transporters (for example Na^+^-K^+^-ATPase) are usually in the micromolar, range [35, 104–106], a millimolar [ATP]_c_ in astrocytes appears to be sufficiently high to reliably fuel the ATP-consuming enzymes and transport processes.

**Table 3:** The cytosolic concentration of ATP ([ATP]_c_) in astrocytes as determined by GEMI imaging

Species	Brain region	Preparation	Sensor/calibration	[ATP]_C_ (mM)	Reference
Rat	Cerebellum	Cell culture	Luciferase/digitonin permeabilization	1.5	[102]
Rat	Hypothalamus	Cell culture	Luciferase/digitonin permeabilization	1.4	[102]
Mouse	Cortex	Cell culture	ATeam dual nanosensor	1.5	[64]
Mouse	Cortex	Acute slice	ATeam dual nanosensor	0.7–1.3	[64]

Astrocytes are well known for their heterogeneity in morphology [107–111] and metabolism [108, 112–119], suggesting that also the ATP content may differ between individual astrocytes or between astrocytes from different brain regions. Cultured astrocytes that are derived from hypothalamus and cortex showed no sensor-detectable differences in basal cytosolic ATP contents [120]. However, imaging of ATP GEMIs has provided evidence for a higher basal [ATP]_c_ in cortical astrocytes (grey matter) compared to astrocytes in the corpus callosum (white matter), thereby emphasizing metabolic heterogeneity of astrocytes across brain regions [121].

## ATP Consumption by Astrocytes

A large number of enzymatic reactions in various biochemical pathways continuously consume ATP in astrocytes (Table 1). The overall consumption of ATP by cultured astrocytes accounts for around 4 nmol/(mg x min), as calculated from the time-dependent loss in cellular ATP content after inhibition of both glycolysis and oxidative phosporylation (by application of 2-deoxyglucose (2DG) and inhibitors of oxidative phosphorylation) [55, 67]. This value matches perfectly to recent data that report a continuous ATP regeneration of 4 nmol/(mg x min) in human induced pluripotent stem cell (iPSC)-derived astrocytes [122]. Therefore, assuming a cellular content of 30 to 40 nmol/mg (Table 2) around 10% − 15% of cellular ATP is consumed and regenerated per minute in non-stimulated cultured astrocytes. Such data have to our knowledge not been reported for stimulated astrocytes or for astrocytes in vivo.

**Fig. 4:**
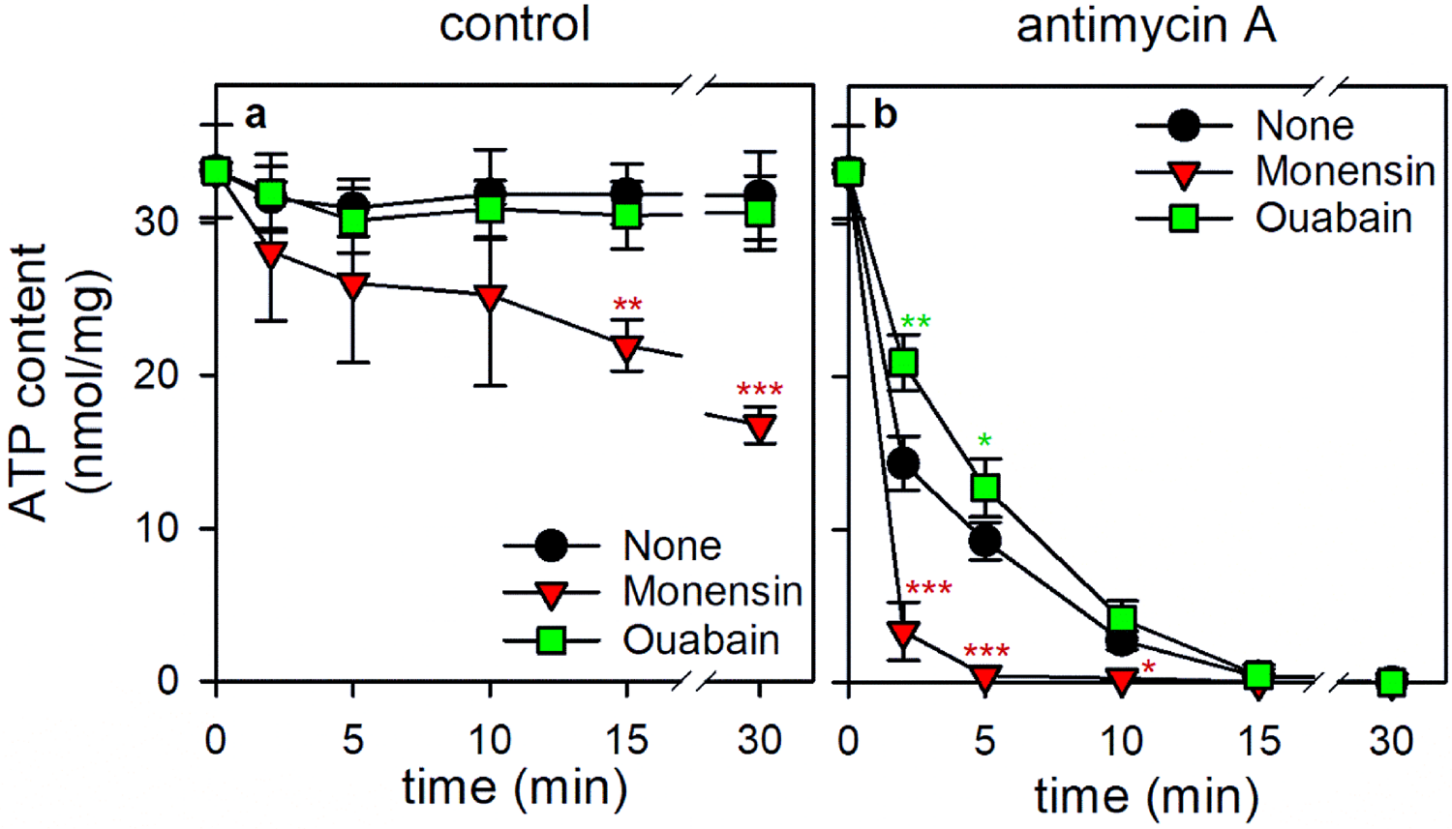
Modulation of ATP consumption in cultured rat astrocytes. Astrocyte cultures were preincubated for 60 min in glucose-free incubation buffer to deplete the cells of glycogen [128]. Subsequently, the cells were incubated in glucose-free incubation buffer [55] without (a) or with (b) the complex III inhibitor antimycin A (1 µM) and in the absence or the presence of the Na^+^-K^+^-ionophore monensin (20 µM) or the Na^+^-K^+^-ATPase inhibitor ouabain (1 mM). After the given incubation periods, the cellular ATP content was determined as previously described [55]. The viability of the cells was not compromised as demonstrated by the absence of any significant increase in extracellular LDH activity during the 30 min incubation compared to the respective control conditions (data not shown). The data shown are means ± SD of values obtained in three experiments performed on independently prepared cultures. The average initial ATP content of the cultures after the preincubation was 35.4 ± 2.3 nmol/mg and the protein content of the cultures was 146 ± 22 µg/well. The significance of differences (ANOVA) compared with the data obtained for the respective control incubation (none) is indicated by *p < 0.05, **p < 0.01 and ***p < 0.001

To which extent the different ATP consuming reactions contribute to the overall ATP consumption in astrocytes has not been explored in detail. There are just too many enzymatic reactions and pathways that consume ATP in astrocytes (Table 1; Fig. 2) to discriminate reliably between the individual contribution of given enzymes in ATP conusmption. In addition, the relative contribution of different ATP consuming reactions will strongly depend on the physiological situation, the activation state and the environment of an astrocyte, consistent with the reported heterogeneity of astrocytes [107, 114, 123, 124]. Reliable data are only available for the strongest consumer of ATP in astrocytes, the Na^+^-K^+^-ATPase [31], which was discussed to consume between 20% and 50% of the continuously regenerated ATP [65, 125], consistent with the important function of Na^+^-K^+^-ATPase in actively buffering extracellular K^+^ that is released by neurons during neurotransmission in brain [9, 30]. The importance of Na^+^-K^+^-ATPase as main consumer of astrocytic ATP is confirmed by recent data from our group (Fig. 4). When astrocytes, after depletion of glycogen, were incubated in the absence of glucose, the ATP content remained high as previously reported [55, 62] and was not affected by the presence of the Na^+^-K^+^-ATPase inhibitor ouabain (Fig. 4a). In contrast, presence of the Na^+^-K^+^-ionophore monensin lowered the cellular ATP content of glucose-deprived astrocytes by around 50% within 30 min (Fig. 4a), suggesting that the monensin-induced accelerated consumption of cytosolic ATP for Na^+^-K^+^-ATPase-mediated ion pumping cannot be compensated by mitochondrial ATP regeneration (Fig. 4a). Inhibition of mitochondrial respiration in glucose-deprived astrocytes by antimycin A caused a rapid decline in cellular ATP content (Fig. 4b) as previously reported [55, 62]. For such conditions, the rapid antimycin A-induced ATP loss was slowed by application of the Na^+^-K^+^-ATPase inhibitor ouabain and further accelerated by the presence of the Na^+^-K^+^-ionophore monensin (Fig. 4b), supporting the prominent contribution of Na^+^-K^+^-ATPase in astrocytic ATP consumption.

The sodium gradient established by the Na^+^-K^+^-ATPase is used to fuel many important transport processes in astrocytes [31], including the Na^+^-dependent uptake of amino acids such as glutamate [126] and the removal of Ca^2+^ from the cytosol [127]. Accordingly, application of glutamate lowers the cytosolic ATP concentration ([ATP]_c_) in astrocytes by a process that depends on the sodium-dependent glutamate uptake [81].

## ATP Regeneration from AMP and ADP

The maintenance of a high steady state cellular ATP content demonstrates that ATP consumed in astrocytes is efficiently and continuously regenerated. Main products of ATP consuming reactions are ADP and AMP (Fig. 2). In untreated astrocytes the contents of these two nucleoside phosphates are rather low and the AEC is very high [55, 60, 66, 69, 72, 82, 83]. Transient increases in the cellular ADP and AMP contents of astrocytes were observed, if both glycolysis and oxidative phosphoylation are impaired [55, 60] as well as in hypoxic astrocyte cultures after reoxygenation [83]. However, the increases found in ADP and AMP contents do not match the loss in cellular ATP content. In fact, the total amount of cellular adenosine phosphates is severely lowered after such treatments, suggesting that accumulating AMP is rapidly metabolized [55, 60], most likely to adenosine and inosine as previously shown for starved C6 glioma cells [129].

**Fig. 5:**
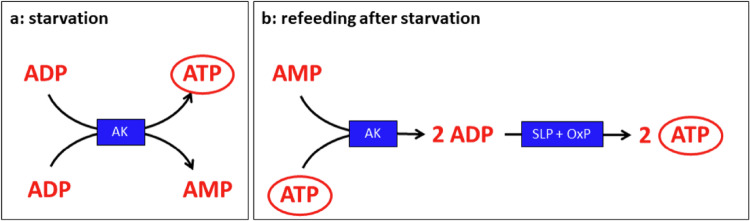
Functions of adenylate kinase (AK) in the ATP metabolism of astrocytes. AK establishes the equilibrium between cellular concentrations of ATP, ADP and AMP. During starvation that leads to ADP accumulation due to insufficient ATP regeneration (a), AK transfers an energy-rich phosphate moiety from one ADP molecule to a second ADP, thereby regenerating ATP and forming AMP. After refeeding following starvation (b), AK phosphorylates AMP to ADP which is subsequently phosphorylated by substrate level phosphorylation (SLP) or oxidative phosphorylation (OxP) to ATP

AMP is the product of enzymatic reactions that activate substrates for biosynthetic processes (Table 1; Fig. 2), is product of the hydrolysis of the second messenger cAMP by phosphodiesterases [56, 130] and is product of the phosphorylation of adenosine by adenosine kinase [38]. During starvation the main producer of AMP is adenylate kinase (Fig. 5a) which makes use of the accumulating ADP to transfer a phosphate group from one ADP substrate molecule to a second ADP, thereby regenerating ATP and forming AMP [57, 131]. Reversely, if sufficient catabolism of energy substrates takes place and ATP is efficiently regenerated from ADP, adenylate kinase phosphorylates AMP by using ATP as substrate (Fig. 5b) and the two ADP molecules generated are efficiently phosphorylated to regain ATP (Fig. 5b). Cultured astrocytes contain substantial activity of adenylate kinase [58] (J. Berger and R. Dringen, unpublished results). This activity is sufficiently high to rapidly reestablish an appropriate equilibrium between the cellular pools of ATP, ADP and AMP as demonstrated by a transient accumulation first of ADP and subsequently of AMP in astrocytes after impairment of ATP regeneration by inhibition of both glycolysis and oxidative phosphorylation [55, 60]. On the other hand, cellular AMP and ADP that had accumulated in ATP-depleted starved astrocytes are rapidly phosphorylated to ATP within 5 min after re-application of glucose [60].

**Fig. 6:**
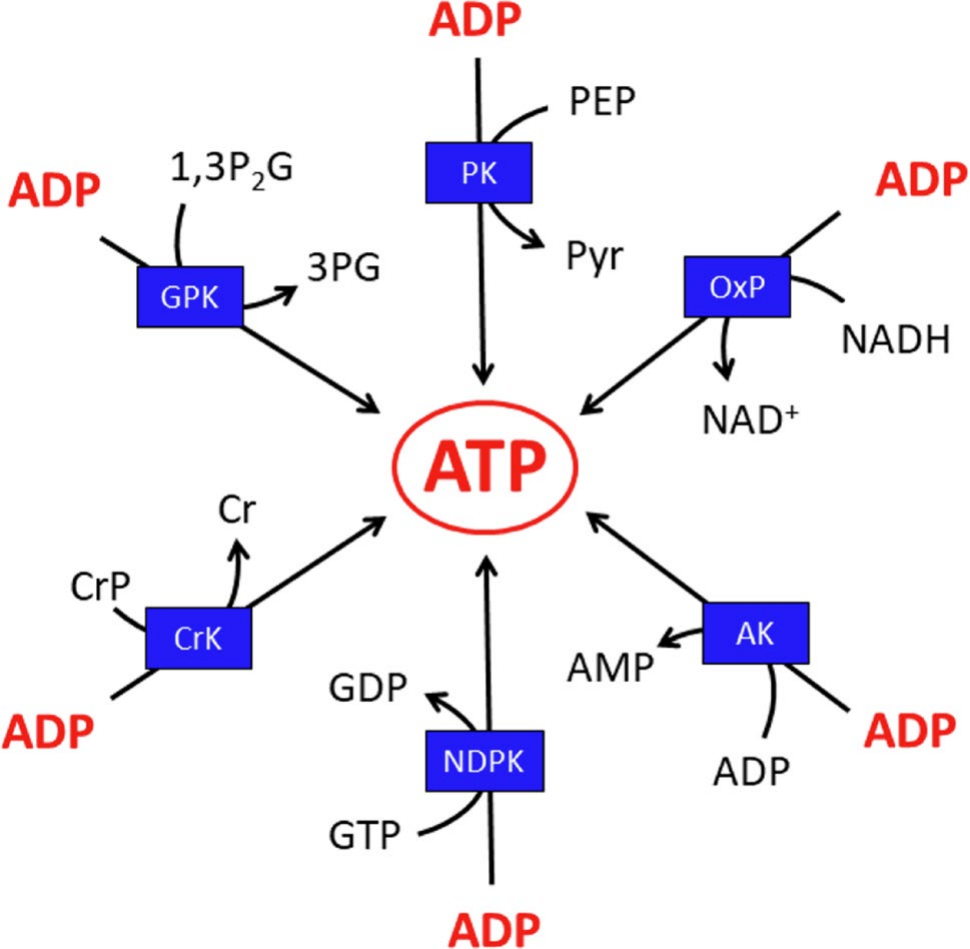
Astrocytic reactions that phosphorylate ADP to ATP. The two ATP regenerating enzymes of glycolysis, 3-phosphoglycerate kinase (GPK) and pyruvate kinase (PK) transfer a high energy phosphate group to ADP from their substrates 1,3-bisphosphoglycerate (1,3P_2_G) and phosphoenole pyruvate (PEP) to generate ATP and the products 3-phosphoglycerate (3PG) and pyruvate (Pyr), respectively. NADH provides electrons for the complex I of the respiratory chain, thereby initiating the mitochondrial oxidative phosphorylation (OxP). Additional electrons for the oxidative phosphorylation are provided by the oxidation of substrates that are catalyzed by other flavine-containing enzymes of the inner mitochondrial membrane. Adenylate kinase (AK) transfers the terminal phosphate group of one ADP molecule to a second ADP molecule, thereby generating ATP and AMP. Nucleoside diphosphate kinases (NDPK) make use of GTP to phosphorylate ADP. Creatine kinase (CrK) uses the high energy phosphate group of creatine phosphate (CrP) to phosphorylate ADP and generate ATP and creatine (Cr). In the presence of a high ATP concentration, the reactions catalyzed by GPK, CrK, NDPK and AK run in the opposite direction to phosphorylate 3PG, Cr, GDP and AMP, respectively

Most of the ADP generated by ATP-consuming reactions (or by adenylate kinase) will be phosphorylated in astrocytes to regenerate ATP. Several enzymes and pathways can contribute to the phosphorylation of ADP in astrocytes (Fig. 6). Quantitatively most important for ADP phosphorylation in astrocytes are the glycolytic enzymes 3-phosphoglycerate kinase (GPK) and pyruvate kinase (PK) in the cytosol and the oxidative phosphorylation in mitochondria (Fig. 6). These pathways have a high capacity in the presence of suitable substrates and contribute both to the ATP regeneration in glucose-fed astrocytes [62, 67, 122]. Aditionally, nucleoside diphosphate kinases (NDPK) can use other nucleoside triphosphates, such as GTP (Fig. 6) that is continuously generated in the citric acid cycle, as substrate to phosphorylate ADP [132].

Also, creatine kinase (CrK) efficiently phosphorylates ADP on the expense of CrP in astrocytes (Fig. 6), especially in situations of a compromised energy metabolism that leads to substantial accumulation of ADP [55, 60]. CrK-mediated phosphorylation is very rapid, but has only the rather low capacity of the existing cellular CrP pool. Thus, CrP is mainly considered as energy buffer for emergency situations [55, 133].

ATP regeneration from ADP has to match the ATP consumption of around 4 nmol/(min x mg) [55, 62, 67, 122] to maintain a high ATP level and a high AEC in astrocytes. Both glycolytic substrate level phosphorylation as well as mitochondrial oxidative phosphoylation contribute to ATP regeneration from ADP and both processes have reserve capacity to compensate at least in part for an impairment of the other process. Data from the simultaneous measurement of extracellular acidification and oxygen consumption revealed that in glucose-fed human iPSC-derived astrocytes glycolysis and oxidative phosphorylation contribute both around 50% to the ATP regeneration and that accelerated glycolysis can compensate for an impairment of mitochondrial ATP regeneration [122]. This is consistent with data from inhibitor experiments performed on cultured rat astrocytes. In the presence of glucose, impairment of mitochondrial respiration lowers the cellular ATP content only slowly and even after hours still around half of the intial ATP content is present in the cells due to an upregulation of glycolytic ATP regeneration as demonstrated by accelerated lactate production [62, 134]. Similarly, if glycolysis and glycogen mobilization are impaired by application of 2DG, the ATP content is lowered within several hours only by around 50% as endogenous energy stores are used for mitochondrial ATP regeneration under such conditions [67].

## ATP Depletion in Astrocytes

The ATP content of astrocytes can be severely lowered by treatments that impair cellular ATP regeneration. This has been reported mainly for extended incubations in the absence of suitable energy substrates or for incubations with inhibitors of the main ATP regenerating pathways. A substantial depletion of cellular ATP contents of astrocytes has severe concequences on astrocytic viability [62, 79, 135] and has been connected with impaired astroglial glutamate uptake [136, 137] and cell swelling induced release of excitatory amino acids [138, 139].

### Impairment of ATP Regeneration by Deprivation of Energy Substrates

The high ATP content of cultured astrocytes is maintained for several hours even in the absence of any exogenous energy substrate [62], suggesting that endogenous energy stores such as fatty acids and glycogen are mobilized by the cells to fuel ATP regeneration during starvation. Indeed, the utilization of such endogenous energy stores is impaired by inhibition of oxidative phosphorylation or simultaneous inhibition of the mitochondrial uptake of pyrvuvate and activated fatty acids [55, 62, 67]. However, the cellular amounts of endogenous energy stores are limited and during starvation periods of more than 8 h the cellular ATP content of cultured astrocytes is lowered. After 24 h of starvation, cultured astrocytes are still viable but contain only around 30% of the initial ATP content [62, 70], while longer incubations cause a further depletion of the cellular ATP content below a threshold level of 25% that is needed for the survival of starved astrocytes.

### Inhibition of ATP Regenerating Pathways

Both glycolytic substrate level phosphorylation and mitochondrial oxidative phosphorylation appear to have in astrocytes substantial reserve capacity for ATP regeneration to compensate at least in part for an impairment of the other pathway. The high potential of glycolysis for ATP regeneration is demonstrated by studies showing that astrocytes survive even an inactivation of the mitochondrial respiratory chain as long as sufficient glucose is available, both in culture [140] and in vivo [141]. In glucose-fed astrocytes, the inhibition of mitochondrial oxidative phosphorylation by antimycin A or an uncoupler such as BAM15 [62] does not lead to a rapid alteration in cellular ATP levels and it takes a few hours before the astrocytic ATP content is severly lowered. A similarly slow ATP loss was also reported for astrocytes that had been incubated under ischemic conditions [68, 79, 142]. However, for inhibition of oxidative phosphorylation by azide, both stable astrocytic ATP contents and rather quickly decreasing levels of ATP have been reported [60, 77, 111].

Inhibited oxidative phosphorylation in glucose-fed astrocytes accelerates glycolysis as demonstrated by increased glucose consumption and lactate production [62, 134, 140]. This compensates for some of the loss of mitochondrial ATP regeneration capacity [122]. Such an upregulation of glycolysis is impossible in glucose-deprived astrocytes and consequently ATP contents decline rapidly, if such astrocytes are exposed to mitochondrial inhibitors such as rotenone, antimycin A, oligomycine [55, 62, 143] or fluorocitrate [144]. Glucose depletion alone has no immediate consequence on the ATP content of cultured astrocytes, as endogenous energy stores are used to fuel mitochondrial ATP regeneration under such conditions [62]. However, inhibition of glycolysis by application of 2DG lowers the cellular ATP content both in glucose-deprived and glucose-fed astrocytes during the initial minutes of incubation by formation and accumulation of 2DG-6-phosphate (2DG6P), but this initial ATP decline slows down and even after hours of incubation with 2DG around half of the initial cellular ATP content is detectable, consistent with efficient ATP regeneration by mitochondrial metabolism [67].

The contribution of both glycolysis and oxidative phosphorylation in ATP regeneration in astrocytes is clearly demonstrated by results from coincubation studies with inhibitors of both pathways. Such treatments which inhibit glycolysis and oxidative phosphorylation severely and rapidly lower the cellular ATP content as shown for incubations of astrocytes with iodoacetate plus azide [64, 73, 81, 135], fluoride plus azide [73], 2DG plus antimycine A [55, 67], 2DG plus rotenone [67, 138], 2DG plus oligomycine [67], 2DG plus cyanide [139] or 2DG plus the uncoupler BAM15 [60, 67].

**Fig. 7:**
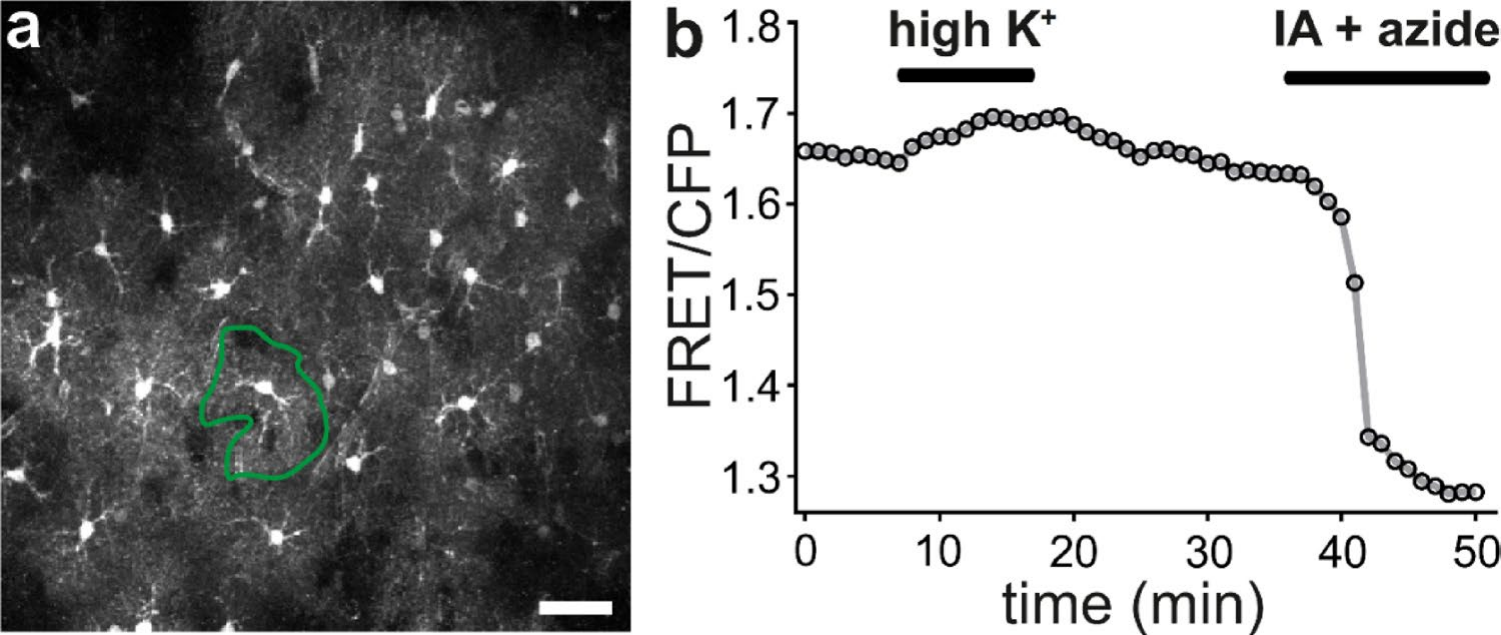
Imaging of ATP in astrocytes in acute brain slices. a: Astrocytes in an acute brain slice of the mouse cortex expressing the ATeam1.03YEMK sensor. The sensor was expressed by adeno-associated virus-mediated gene transfer. Shown is the FRET channel imaged by 2-photon-laser-scanning microscopy in an acute brain slice (scale bar: 20 μm). b: Trace of the FRET/CFP ratio of the cell outlined by the green line in panel a. After recording a stable baseline, the concentration of K^+^ was increased from 2.5 mM to 10 mM for the time indicated resulting in an increase in the ATP sensor signal. At the end of the experiment, ATP production was blocked by application of iodoacetate (IA, 1 mM) and sodium azide (10 mM). Cellular ATP consumption in the absence of ATP regeneration results in a decrease of the ATP sensor signal to a level reflecting the absence of detectable ATP in the cell. For experimental details see [121]

The quantitative data mentioned above on a depletion of the total specific content of ATP in cultured astrocytes by application of inhibitor of ATP regenerating pathways have been confirmed by ATP sensor studies showing a corresponding decline in the cytosolic ATP content of astrocytes in different model systems (Table 4). For example, inhibition of glycolysis and oxidative phosphorylation by coapplication of iodoacetate plus azide caused a rapid decrease of the ATP sensor signal in astrocytes of acute mouse cortical slices to a level reflecting the absence of detectable ATP in the cell (Fig. 7b).

**Table 4:** Modulation of ATP content of astrocytes in different model systems assessed by GEMIs for cytosolic ATP

Species	Brain area/Experimental system	Treatment	Consequence on the ATP signal/ Δ[ATP]_c_ *	Reference
Mouse	Cortex/ Mixed cultures	Iodoacetate	Decrease	[145]
Mouse	Cortex/ Mixed cultures	Azide	Decrease	[146]
Mouse	Cortex/ Astrocyte culture	Azide	Decrease^1^	[64]
Mouse	Cortex/ Astrocyte culture	Azide + Iodoacetate	Strong decline	[81]
Human	iPSC-derived astrocytes	2-Deoxyglucose	Decrease	[147]
Human	iPSC-derived astrocytes	Mitochondrial uncoupler FCCP	Decrease	[147]
Mouse	Cortex/ Organotypic slice culture	Chemical ischemia	Reversible decrease	[142, 148]
Mouse	Motor Cortex/ In vivo	Fluorocitrate	Decrease	[149]

This table summarizes data from selected references. *Differences in cytosolic ATP concentrations (Δ[ATP]_c_) for a given treatment are only available for studies that have quantified these concentrations from the determined changes in sensor signals. The reported Δ[ATP]_c_ values are: ^1^Decrease of Δ[ATP]_c_ by 0.5 mM (−32%)

## Monitoring Alterations of Cytosolic ATP Content in Stimulated Astrocytes

ATP GEMIs expressed in astrocytes were used to study the dynamics of [ATP]_c_ in different preparations including cultured astrocytes, organotypic slice cultures and acute brain slices (Table 5). Several studies report that [ATP]_c_ decreases during application of glutamate to astrocytes [64, 81, 121, 150], consistent with glutamate uptake via the sodium-dependent glutamate transporters EAAT1 or EAAT2 (also called GLAST and Glt-1, respectively), followed by Na^+^-extrusion via Na^+^-K^+^-ATPase [151]. In addition to glutamate, K^+^ is released from active neurons during repolarization (also from non-glutamatergic neurons); therefore, an increased potassium concentration in the extracellular space ([K^+^]_e_) is considered an universal sign of neuronal activity. Strikingly, several studies consistently report that an increase of [K^+^]_e_ results in increased astrocytic [ATP]_c_ (Table 5), at least immediately after the rise of [K^+^]_e_ (Fig. 7) [64, 81, 121, 150, 152, 153]. This finding implies that astrocytes contain a substantial amount of ADP under basal conditions, which can be rapidly phosphorylated to ATP when [K^+^]_e_ increases. Consistently, an increase of [K^+^]_e_ stimulates glycolysis [154], thereby providing a metabolic explanation for the observed increased [ATP]_c_ despite the higher ATP demand by Na^+^-K^+^-ATPase. Most interestingly, application of both glutamate and increased [K^+^]_e_ resulted in elevated [ATP]_c_ in astrocytes in acute brain slices obtained from cortex and corpus callosum, suggesting that the K^+^ -induced stimulation of ATP regeneration outperforms increased ATP consumption induced by glutamate uptake [121]. However, using the GEMI PercevalHR, a decrease of the ATP/ADP ratio has been reported in astrocytes challenged with a very high (50 mM) concentration of K^+^ [155]. While this observation theoretically might be explained by a larger increase of ADP despite an increase of ATP, a mechanistic explanation how both ATP and ADP increases simultaneously within minutes is lacking, suggesting that these observations might rather be due to technical differences compared to the experiments performed with the ATeam-type of GEMIs, which report the concentration of ATP. Furthermore, in a model of high intrinsic network activity in hippocampal organotypic brain slices, a short increase (2 min) of [ATP]_c_ by 0.13 mM was observed in astrocytes, which was followed by a decrease by 0.22 mM and 0.37 mM after 5 min and 10 min, respectively [103]. In contrast, in neurons [ATP]_c_ dropped immediately [103]. This finding has been corroborated in acute slices showing that [ATP]_c_ decreases in astrocytes both in the cortex and corpus callosum after 10 min of epileptic form activity [121]. While it is reasonable to assume that during network activity both glutamate and K^+^ increase in the environment of astrocytes, the precise timing and/or concentration-dependencies of the effects of the different signals particularly in the brain in vivo needs further investigation. Finally, the impact of insulin and leptin signaling on the metabolism of astrocytes and specifically on ATP levels has been studied using ATeam GEMIs as well as Perceval HR, reporting a slight decrease of [ATP]_c_ as well as of the ATP/ADP ratio [120, 155].

Analysis of the dynamics of [ATP]_c_ in astrocytes using GEMIs has revealed important new insights in metabolism of these cells and its regulation. However, the full potential of this methodology to study astrocytes awaits further application, especially regarding analysis in vivo. For the analysis of ATP in neurons a transgenic mouse line expressing the ATP sensor ATeam1.03^YEMK^ in neurons is available [156], which has been used to image [ATP]_c_ in neurons in situ and in vivo [157–161]. However, a corresponding mouse line for expression in astrocytes is currently lacking, thus requiring the expression of ATP GEMIs via viral transfer. Nevertheless, given the major advantageous properties of GEMIs, these sensors will certainly contribute to study metabolic properties of astrocytes in the future.

**Table 5:** Modulation of the cytosolic ATP concentration in stimulated astrocytes in different model systems assessed by ATP sensors

Species	Brain area/Experimental system	Treatment	Alteration in ATP signal/Δ[ATP]_c_ *	Reference
**Cell cultures**				
Mouse	Cortex/ Astrocyte cultures	Glutamate	Decrease^1^	[64]
Rat	Hippocampus/ Mixed cultures	Glutamate	Decrease of ATP/ADP ratio	[155]
Mouse	Cortex/ Astrocyte cultures	Dopamine	No change	[81]
Mouse	Cortex/ Mixed cultures	Elevated [K^+^]	Increase	[150]
Mouse	Cortex/ Astrocyte cultures	Elevated [K^+^]	Increase^2^	[64]
Rat	Hippocampus/ Mixed cultures	Strongly elevated [K^+^] (50 mM)	Decrease of ATP/ADP ratio	[155]
Mouse	Cortex, Hypothalamus/ Astrocyte cultures	Insulin	Decrease	[120]
Mouse	Cortex, Hypothalamus/ Astrocyte cultures	Leptin	Decrease	[120]
**Organotypic slice cultures**				
Mouse	Cortex	Recurrent network activity	Initial increase^3^	[103]
Mouse	Hippocampus	Glutamate	Decrease	[150]
Mouse	Hippocampus	Elevated [K^+^]	Increase	[150, 152, 153]
**Acute brain slices**				
Mouse	Cortex, corpus callosum	Glutamate	Decrease	[121]
Mouse	Cortex, corpus callosum	Elevated [K^+^]	Increase	[121, 152]
Mouse	Cortex, corpus callosum	Glutamate + elevated [K^+^]	Increase	[121]
Mouse	Cortex, corpus callosum	Intrinsic network activity	decrease	[121]

This table summarizes data from selected references. *Differences in cytosolic ATP concentrations (Δ[ATP]_c_) for a given treatment are only available for studies that have quantified these concentrations from the determined changes in sensor signals. The reported Δ[ATP]_c_ values are: ^1^Decrease of Δ[ATP]_c_ by 0.16 mM (−11%); ^2^Increase of Δ[ATP]_c_ by 0.07 mM (+ 5%); ^3^Initial increase of Δ[ATP]_c_ by 0.13 mM followed by a decrease of Δ[ATP]_c_ of 0.22 to 0.37 mM

## Endogenous Energy Stores that Can Fuel Astrocytic ATP Regeneration

For starved astrocytes, three endogenous energy stores have to be considered to fuel ATP regeneration from ADP during insufficient supply of exogenous substrates, CrP, glycogen and lipids. The cellular CrP pool allows very rapid ATP regeneration, but has only a very low quantitative capacity due to the similar specific contents of CrP and ATP in astrocytes. Both glycogen and fatty acids have a much stronger potential to fuel ATP regeneration. Glycogen is rapidly mobilized during starvation and from one glucose molecule in glycogen more than 30 ATP molecules can be regenerated by complete oxidation of a glucose 6-phosphate molecule via glycolysis and mitochondrial oxidation. Fatty acids stored in lipid droplets would even yield more ATP regeneration per fatty acid molecule than glucose. Complete oxidation of palmitate via β-oxidation and mitochondrial oxidation of the generated acetyl-CoA would allow to regenerate more than 100 ATP molecules per fatty acid molecule.

### Creatine Phosphate

In high energy demanding cell types, CrP acts as an immediately available energy substrate that is used by CrK to phosphorylate ADP to ATP [162–165]. For cultured astrocytes, the cellular level of CrP has been reported as similar or even higher than the cellular content of ATP [55, 71, 79, 80, 166]. The CrP content of cultured astrocytes depends on the medium composition [55, 166, 167], the metabolic condition of the cells [55, 79, 166] as well as the culture age [55]. An incubation of cultured astrocytes with creatine doubles the specific content of CrP, but does not affect the cellular ATP content [55].

CrP is generated by phosphorylation of cellular creatine by the enzyme CrK [168]. In astrocytes, the creatine used as substrate of CrK can either be derived from the uptake of extracellular creatine by an active transport process [169] or from *de novo* synthesis of creatine from the precursor amino acids, glycine, arginine and methionine [170, 171].

Cultured astrocytes contain large amounts of CrK with specific activities calculated as high as 3.5 U/mg [58] or 2 U/mg (J. Berger and R. Dringen, unpublished results). This CrK activity is orders of magnitude higher than the overall basal ADP phosphorylating activity of 4 nmol/(min x mg) (i.e. 0.004 U/mg) of glycolysis plus oxidative phosphorylation [62, 67, 122], consistent with literature data reported for other cell types [172]. The high activity of CrK in astrocytes maintains a high content of CrP as long as the AEC is high [55].

CrP serves as rapidly accessible buffer for high energy-rich phosphates to prevent severe ATP depletion under conditions of acutely accelerated ATP demand. Especially for conditions of impaired ATP regeneration, a high cellular CrP content enables astrocytes to rapidly regenerate ATP by phosphorylation of ADP on the expense of their CrP content [55, 79, 166]. Accordingly, the loss in cellular CrP level in astrocytes always precedes a loss in cellular ATP content [55].

Of the two main isoforms of CrK, the cytosolic brain-type CrK and the mitochondrial CrK [168], astrocytes contain predominantly the cytosolic isoform and only little mitochondrial CrK [173]. Also, in the human cortex strong immunoreactivity for cytosolic brain-type CrK was found particularly in astrocytes [36], suggesting that this enzyme plays an important role in managing the CrP pool as energy buffer in astrocytes.

### Glycogen

Glycogen is a branched glucose polymere that is stored in healthy brain mainly in astrocytes and will be degraded in hypoglycemic conditions [47, 158, 174]. The glycosyl moieties of glycogen can be quickly mobilized to glucose-1-phosphate by phosphorolysis, catalyzed by glycogen phosphorylase in a process that consumes inorganic phosphate as substrate and does not require activation by or consumption of ATP. During glycogen mobilization, only the hydrolysis of glucose from the branches by the debranching enzyme liberates some free glucose that has to be activated to glucose 6-phosphate by hexokinase for further cellular metabolism. Disturbances in brain glycogen metabolism have been connected with a range of human disorders [47, 175–178], underlying the importance of glycogen in astrocytes as energy store that can be mobilized to fuel glucose-dependent processes.

Cultured astrocytes contain a specific glycogen content of around 70 nmol glycosyl residues per mg of protein [179, 180]. During glucose deprivation the astrocytic glycogen is rapidly mobilized and the glycogen content declines with a half-time of 7 min, lowering cellular glycogen contents to around 10 nmol/mg within 20 min of incubation [179]. During starvation, most of the glycogen-derived glucose phosphate is metabolized via glycolysis to lactate that is exported from the cells [128]. Thus, around 60 nmol glucose/mg are mobilized within 20 min in glucose-deprived astrocytes, mainly by phosphorolysis. Assuming that the main product of glycogen mobilization is already phosphorylated glucose, around 3 ATP can be regenerated from ADP during glycolytic breakdown from each glucose moiety mobilized from glycogen. Thus, exclusive glycolytic metabolism of the 60 nmol/mg glucosyl residues mobilized from glycogen to lactate [128] during 20 min starvation would regenerate 180 nmol/mg ATP, thereby having then potential to fuel 6 times a complete regeneration of the specific cellular ATP pool (around 30 nmol/mg) of cultured astrocytes and to supply the basal ATP consumption (4 nmol/(min x mg)) for 45 min. In contrast, the total oxidation of glycogen-derived carbon atoms in astrocytes via glycolysis plus mitochondrial metabolism would provide around 30 nmol ATP per glucose moiety, enabling the cells to potentially fuel the basal cellular ATP consumption for 450 min.

2DG is frequently applied to cultured astrocytes to inhibit glycolysis. This compound is rapidly phosphorylated to 2DG6P which accumulates in the cells [67]. As the phosphorylation of 2DG by hexokinase consumes ATP, the specific ATP content of 2DG-treated astrocytes declines rapidly to some extent [67, 138]. In addition, as 2DG6P is a potent inhibitor of glycogen phosphorylase [180], the mobilization of glucose from glycogen and subsequenty the use of glycogen-derived glucose phosphate for ATP production is prevented during 2DG exposure. A 2DG-treatment will mainly affect the glycolytic ATP regeneration in astrocytes, while oxidative phosphorylation from other endogenous energy sources allows at least partial maintenance of ATP in 2DG-treated astrocytes [67]. The 2DG-induced accelerated decline of cellular ATP in glucose-deprived astrocytes that were exposed to inhibitors of mitochondrial phosphorylation [67] demonstrates that indeed glycolytic metabolism of glycogen-derived glucose phosphate contributes substantially to the glycolytic ATP regeneration in glucose-deprived astrocytes at least during the initial phase of starvation.

### Lipids

Astrocytes are well known for their ability to metabolize exogenous fatty acids via mitochondrial β-oxidation and to subsequently utilize fatty acid-derived acetyl-CoA for oxidation to CO_2_ in the mitochondrial citric acid cycle or for ketogenesis, both in culture [147, 181–184] and in brain slices [185, 186]. Accordingly, applied long and middle chain fatty acids are able to maintain the high ATP content in glucose-deprived astrocytes [70].

Due to their ability to use endogenous energy substrates to prevent a rapid loss in cellular ATP content, cultured astrocytes can survive a 24 h starvation period in the absence of exogenous substrates [62]. This ability of astrocytes is impaired by the application of etomoxir, an inhibitor of carnitine palmitoyltransferase I [187, 188], which is involved in the uptake of activated long-chain fatty acids from the cytosol into mitochondria, demonstrating that astrocytes indeed use endogenous fatty acids to fuel ATP regeneration.

A likely source of the endogenous fatty acids in starved astrocytes are lipid droplets (LDs). LDs contain a core of neutral lipids (including triacylglycerols) that is surrounded by a phospholipid monolayer. LDs are found in brain mainly in glial cells [189]. In cultured astrocytes, LDs have an average diameter of 450 nm and are localized mainly in the cytosol [190]. Under stress conditions, astrocytes accumulate more LDs which has been discussed as response to better support energy metabolism and to provide protection against stress-induced lipotoxicity [190]. So far, little is known on the functions of LDs in astrocytes but this research area is considered as an important topic for future research [189, 190]. In vivo, another source of fatty acids could be myelin as it has recently been shown that myelin is degraded under energy starvation conditions to maintain energy homeostasis in brain cells [191].

## Use of Extracellular Substrates to Maintain a High ATP Content in Astrocytes

Cultured astrocytes maintain a high cellular ATP content even in the absence of any exogenous energy substrate for at least 8 h by making use of their endogenous energy stores [62]. However, these endogenous energy sources are limited and during a longer starvation period of 24 h the cellular ATP content declines to around 30% of the initial high value [89]. This loss of cellular ATP during starvation can be prevented by the presence of a variety of suitable exogenous energy substrates [62, 70] including hexoses, monocarboxylates, fatty acids, amino acids and nucleosides (Table 6). The pathways involved in the utilization of the substrates that were found to maintain a high ATP content in glucose-deprived astrocytes (Table 6) have previously been discussed [62, 70].

**Table 6:** Exogenous substrates that maintain a high ATP content in glucose-deprived cultured astrocytes

Substrate classes	Substances
Hexoses	mannose, fructose
Monocarboxylates	lactate, pyruvate, β-hydroxybutyrate, acetate
Fatty acids	octanoate, decanoate, palmitate
Amino acids	glutamate, glutamine, alanine, aspartate, lysine, proline
Nucleosides	adenosine, inosine, guanosine

The given exogenous substrates were found to prevent a loss in the cellular ATP content in cultured astrocytes during a 24 h incubation in the absence of glucose [62, 70]

The ability of an exogenous substrate to maintain a high ATP content in glucose-deprived astrocytes demonstrates that uptake and cellular metabolism of the respective substrate are sufficiently fast to fuel substantial amounts of oxidizable carbon into astrocytic metabolism. Analysis of the concentration-dependency of the ATP maintaining effects of the investigated exogenous substrates revealed that such substrates have quite different potentials to fuel astrocytic ATP regeneration during starvation [70]. If the different number of oxidizable carbon atoms in the exogenous substrates investigated are considered for the comparison, exogenous fatty acids as well as the amino acid proline were found to have a higher potential per oxidizable carbon to prevent ATP loss during starvation than monocarboxylates, nucleosides or the other amino acids investigated [70]. Proline as well as fatty acids have even a higher potential to maintain a high ATP level in starved astrocytes than glucose [70]. The catabolism of both proline and of activated fatty acids is exclusively mitochondrial, demonstrating the importance of mitochondrial ATP regeneration in astrocytes as well as the ability of these cells to efficiently use exogenous mitochondrial substrates to fuel ATP regeneration in situations of limited glucose supply.

## Export and Extracellular Processing of ATP by Astrocytes

**Fig. 8:**
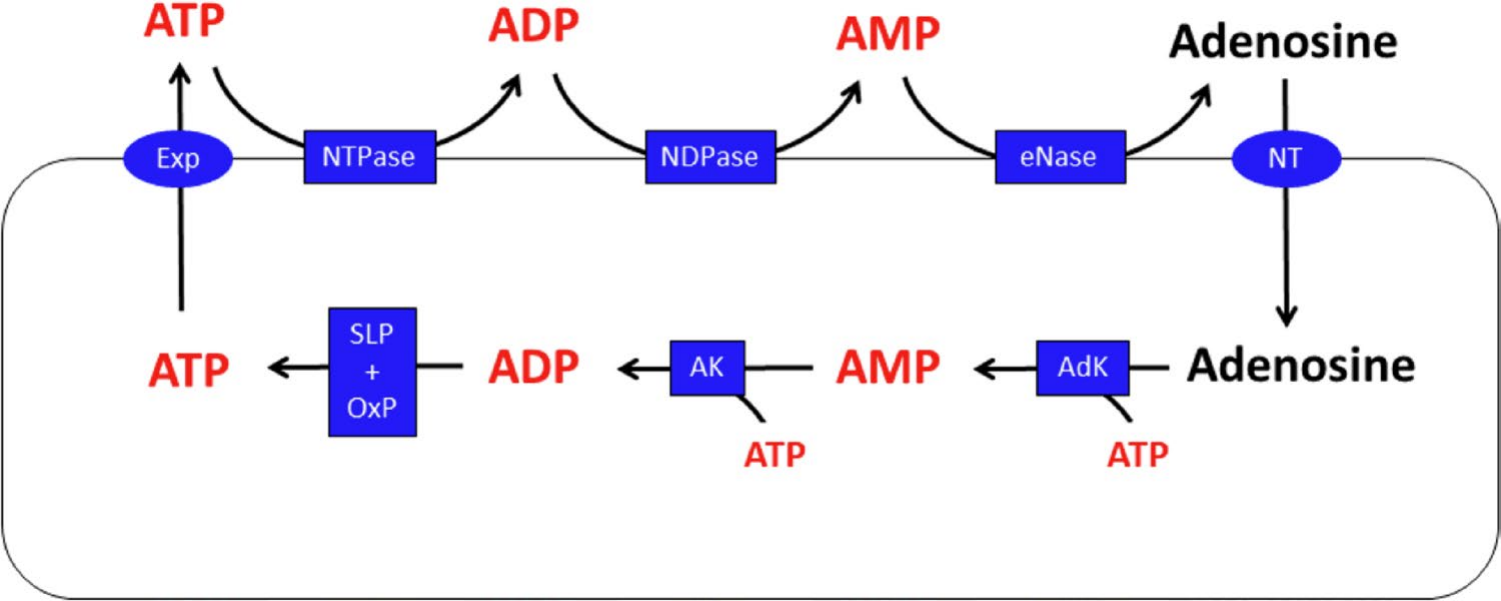
Extracellular ATP processing and cellular utilization of adenosine for ATP restoration in astrocytes. ATP can be exported from astrocytes via different mechanisms (Exp). Extracellular ATP is processed by ectohydrolases (NTPase, nucleoside triphosphatase; NDPase, nucleoside diphosphatase; eNase, ectonucleotidase) to adenosine that is taken up by nucleoside transporters (NT). In astrocytes, adenosine is phosphorylated by adenosine kinase (AdK) to AMP that is further phosphorylated by adenylate kinase (AK) to ADP which is finally phosphorylated via substrate level phosphorylation (SLP) or oxidative phosphorylation (OxP) to restore ATP

In addition to its function as the central cellular energy carrier, ATP acts as an important extracellular signaling molecule [192–194]. ATP is released from cells via several different mechanisms and is rapidly degraded in the extracellular space by different enzymes yielding ADP, AMP and finally adenosine (Fig. 8). ATP and ADP can activate P2-type purinergic receptors, while adenosine activates P1-type purinergic receptors [195]. Thus, purinergic signaling in brain involves a complex interplay between ATP release, expression and activity of enzymes catalyzing the extracellular ATP processing, and presence of the different types of receptors on different types of target cells.

Several different mechanisms have been proposed to mediate ATP release from cells including astrocytes [196]. Astrocytes contain vesicles as well as the appropriate machinery to release the vesicular content in an activity dependent manner [197]. ATP can be loaded into such vesicles by the vesicular nucleotide transporter (VNUT, Slc17A9). For astrocytes, it has been suggested that the ATP-loaded vesicles are lysosomes [198]. From such vesicles ATP is released to the extracellular space in a Ca^2+^ dependent manner as “gliotransmitter” [199]. Besides this vesicular mechanism, ATP can also be released via a number of different channels, including the P2X7 receptor, the connexins Cx26, Cx32 and Cx43, pannexins, VRAC (LRRC8a), CalHM1-3, and the maxi anion channels (Slco2a1) [200]. The release via these channels can be triggered by different stimuli, including membrane voltage, CO_2_, Ca^2+^, pH, mechanical stimulation via Piezo or TRPV4 channels, osmotic stress and ATP itself [200]. Furthermore, also damaged cells release ATP, most likely in rather large amounts. Consistently, processes of microglia cells, which are the resident immune cells of the brain, are strongly attracted by ATP [201].

An interesting aspect of ATP dependent signaling is the potentially direct connection of the cellular energy state ([ATP]_c_ and/or the cytosolic ATP/ADP ratio) and the ATP mediated signaling to other cells. However, it is currently unresolved whether [ATP]_c_ has a direct effect on the amount of ATP released as signaling molecule. In respect to vesicular release, it appears unlikely that filling of vesicles with ATP by VNUT is regulated by [ATP]_c_ (at least for the physiological concentration range), given that VNUT is an active transporter and [ATP]_c_ is rather high. For channel mediated ATP-release, a mathematical model has recently been presented, indicating that reduction of [ATP]_c_ from 3 mM to 2 mM does hardly affect the concentration of ATP released during a stimulus [200]. Even reduction of [ATP]_c_ to 1 mM reduced the ATP released by about 15%, but only under conditions of very slow ATP production [200]. Therefore, it seems unlikely that the release of ATP might be a direct signal of cells to inform their neighbors about the energy status, at least not via this simple concentration dependent mechanism within a physiological concentration range. However, during episodes of severe ATP depletion ATP release might be reduced. Whether such a mechanism could play a role during energy deprivation of the brain, like e.g., during stroke, to regulate brain energy homeostasis, remains to be investigated.

**Fig. 9:**
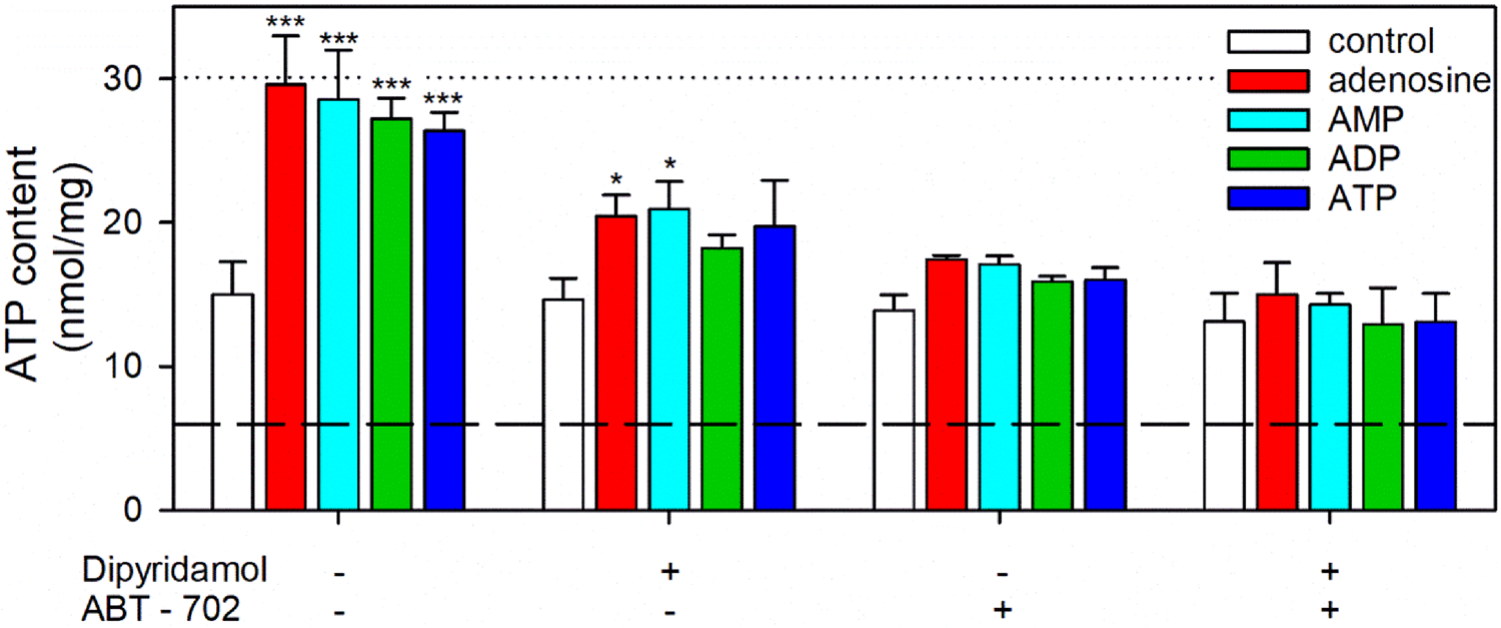
Use of exogenous adenosine phosphates as precursor for cellular ATP restoration in ATP-depleted astrocytes. Primary rat astrocyte cultures were preincubated for 60 min in glucose-free incubation buffer with 1 µM of BAM15 to lower the cellular ATP content [60] before the cells were incubated in glucose (5 mM) containing incubation buffer [60] without or with 100 µM adenosine, AMP, ADP or ATP in the absence or the presence of the nucleoside transporter inhibitor dipyridamol (10 µM) and/or the adenosine kinase inhibitor ABT-702 (10 µM). After 60 min, the cellular ATP content and the cell viability were determined as previously described [55]. The viability of the cells was not compromised as demonstrated by the absence of any increase in extracellular LDH activity (data not shown). The data shown are means ± SD of values obtained in three experiments performed on independently prepared cultures. The average initial ATP content of the cultures (30.0 ± 1.5 nmol/mg) is indicated by the black dotted line and the ATP content after 60 min preincubation was 6.6 ± 1.5 nmol/mg as indicated by the dashed line. The protein content of the cultures was 171 ± 14 µg/well. The significance of differences (ANOVA) compared with the data obtained for the control incubation (absence of adenine-containing substrates and inhibitors) is indicated by *p < 0.05 and ***p < 0.001

Once released, ATP is degraded in the extracellular space by a number of different enzymes, including the ectonucleotidases of the E-NTPDase family (NTPDase1 to 6; also named CD39, CD39L1, CD39L3, UDPase, CD39L4, CD39L2, respectively), of the E-NPP family (NPP1, NPP2, NPP3), alkaline phosphatase, and ecto-5’ nucleotidase, finally yielding adenosine [202]. All the hydrolyzing ectoenzymes needed to process extracellular ATP to adenosine (Fig. 8) are expressed by astrocytes and some of these are even upregulated by the presence of extracellular ATP [203]. Efficient astrocytic hydrolysis of extracellular ATP, ADP or AMP has been reported for cultured astrocytes [204] and these processes are also underlying the ability of cultured astrocytes to use such exogenous adenosine phosphates as substrate for intracellular ATP restoration in ATP-deprived astrocytes (Fig. 9). As this ATP restoration was strongly impaired by inhibitors of nucleoside transporters and adenosine kinase (Fig. 9), it can be concluded that the applied adenosine phosphates had been hydrolyzed to adenosine that was subsequently taken up and phosphorylated by adenosine kinase to the new AMP needed for ATP restoration.

The expression and activity of the ectoenzymes involved in ATP degradation in brain might vary between brain regions, cell types and even very locally on different parts of cellular processes. In combination with the different pathways of ATP release from astrocytes, but also from other cell types in the brain, this extracellular processing results in complex dynamics of ATP and its metabolites within a local environment, which will require a detailed analysis with high temporal and spatial resolution [59].

Extracellular ATP as well as adenosine are important signaling molecules which act as activators of purinergic receptors [59, 195]. Adenosine is an agonist on P1 receptors (also called adenosine receptors). All four types of P1 receptors (A1, A2A, A2B, A3) are G-protein coupled receptors, affecting the cAMP concentration in cells either via G_s_ or G_i_ coupling [195]. P2X receptors are non-selective cation channels which are activated by ATP. Seven types of P2X receptors are known (P2X1 to P2X7) which differ in their properties, e.g. in their affinity for ATP [195]. In contrast, the eight members of the P2Y receptor family (P2Y_1_, P2Y_2_, P2Y_4_, P2Y_6_, P2Y_11_, P2Y_12_, P2Y_13_ and P2Y) are G-protein coupled receptors [195]. Some of these receptors are less specific for ATP, but can also be activated by ADP, UTP or UDP. Astrocytes express all known P2Y receptors, which contribute to regulating many aspects of astrocyte physiology, including Ca^2+^ signaling, neurovascular coupling, gliotransmitter release and cell proliferation [205]. P2Y receptor signaling also plays a crucial role for the activation of astrocytes to reactive astrocytes in disease states [205]. Signaling via these receptors typically induces Ca^2+^ signals in astrocytes, which in turn activate many different types of cellular responses [205, 206]. In summary, the highly diverse receptors and their differential expression patterns add another level of complexity to ATP signaling in the brain.

Signaling by ATP released from astrocytes and/or signaling via purinergic receptors on astrocytes have been implicated in a large variety of physiological functions, including the coordination of synaptic networks [196, 199], the modulation of synaptic plasticity [207], as well as the modulation of phasic and tonic inhibitions in the neocortex [208]. Release of ATP from astrocytes is involved in dopamine-evoked synaptic regulation [209]. Also, neuron-glia assemblies and the intimate cooperation of neurons and glial cells is, at least in part, coordinated by purinergic signaling [210, 211]. Here, not only signaling from astrocytes to neurons occurs, but also from astrocytes to other glial cells [211]. For instance, Ca^2+^ signals in NG2 glia cells are modulated by ATP released from astrocytes [212]. Interestingly, ATP released from astrocytes may spread over about 50–250 µm^2^, thus affecting many synapses in this area [213], a phenomenon also referred as volume transmission [200].

Astrocyte-mediated purinergic signaling including release of ATP plays an important role in signaling in sensory systems like the retina and the olfactory bulb [214]. It also crucially contributes to sensing of physiological parameters in the body and regulates the appropriate physiological responses. Astrocytes sense mechanical stimuli, which might be relevant to detect blood flow and blood pressure [215]. Consistently, they are involved in regulation of blood flow [216] and, therefore, in the BOLD fMRI response [217] as well as in the modulation of cardiorespiratory reflexes [218]. Furthermore, purinergic signaling of astrocytes contributes to regulation of the extracellular pH in the brain [219], oxygen sensing [220], CO_2_ and pH sensing [221], and control of breathing [222–224]. Astrocytic release of ATP is also required for proper glucose homeostasis and insulin signaling. In mice lacking glucose transporter 1 (GLUT1) in astrocytes, insulin-induced release of ATP is augmented and these mice are metabolically healthier [225] and show enhanced glucose metabolism and higher resilience to stroke [226]. Astrocyte-derived adenosine is crucially involved in the modulation of sleep homeostasis [227, 228]. Furthermore, ATP signaling from astrocytes has been implicated in learning and memory consolidation [229]. As astrocytes play an important role in ATP release, extracellular ATP degradation and in adenosine metabolism [59, 230], this brain cell type should be more strongly considered as potential target for treatments of conditions and diseases that have been connected with disturbances in purinergic signalling in brain.

## Restoration of ATP by ATP-Depleted Astrocytes

While depletion of ATP has frequently been studied for cultured astrocytes, only a few reports have addressed astrocytic ATP restoration following an ATP depletion [60, 68, 231, 232]. ATP restoration strongly depends on the availability of energy substrates, but it also requires the availability of a suitable adenine moiety as substrate for the synthesis of new AMP that is needed due to the loss in the total pool of adenosine phosphates that is connected with severe ATP depletion [55, 60]. Pathways that can contribute to the formation of new AMP involve the purine *de novo* synthesis and the purine salvage pathway [4, 233–236]. The purine salvage pathway for AMP synthesis makes use of the free purine bases adenine and hypoxanthine. These bases can be derived from their respective nucleosides, adenosine and inosine.

**Fig. 10:**
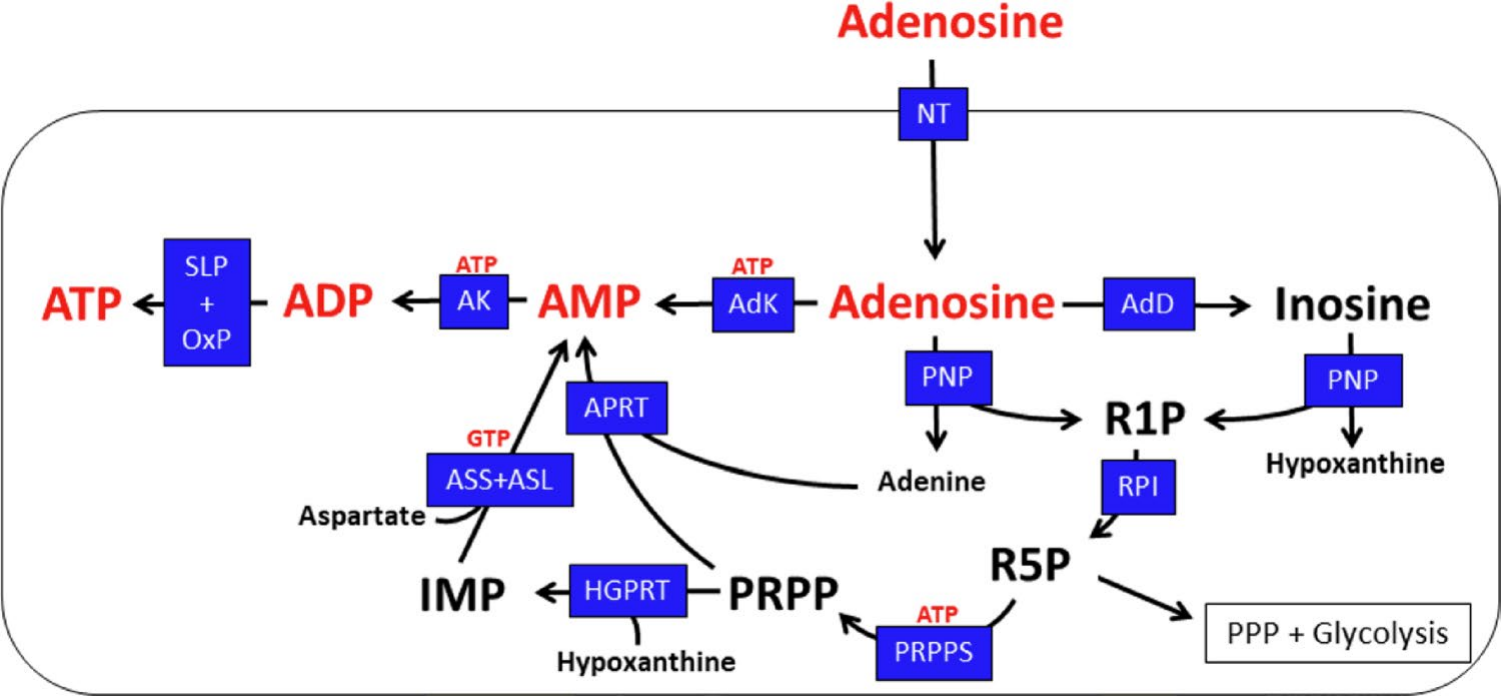
Adenosin metabolism and cellular ATP restoration in astrocytes. AdD, adenosine deaminase; AdK, adenosine kinase; APRT, adenine phosphoribosyl transferase; AK, adenylate kinase; ASL, adenylosuccinate lyase; ASS, adenylosuccinate synthetase; HGPRT, hypoxanthine-guanine phosphoribosyl transferase; IMP, inosine monophosphate; NT, nucleoside transporter(s); OxP, oxidative phosphorylation; PNP, purine nucleoside phosphorylase; PPP, pentose-phosphate pathway; PRPP, phosphoribosyl pyrophosphate; PRPPS, phosphoribosyl pyrophosphate (PRPP) synthase, R1P, ribose-1-phosphate; R5P, ribose-5-phosphate; RPI, ribose phosphate isomerase; SLP, substrate level phosphorylation

Astrocytic sources of these nucleosides are extracellular adenosine that has either been released from brain cells or has been generated by extracellular processing of exported ATP via the activity of astrocytic ectohydrolases [203] via ADP and AMP (Fig. 8). Adenosine can be efficiently taken up into astrocytes by nucleoside transporters [237], subsequently be phosphorylated by adenosine kinase [38, 238], or deaminated to inosine by adenosine deaminase [239] (Fig. 10).

After cellular formation or uptake of adenosine or inosine, the purine base of the nucleoside can be liberated by purine nucleoside phosphorylase that generates in addition to the free bases also ribose-1-phosphate (Fig. 10). The latter can be isomerized to ribose-5-phosphate and further metabolized by the non-oxidative part of the pentose-phosphate pathway (PPP) and glycolysis to gain energy. Alternatively, ribose-5-phosphate can serve as substrate for the generation of phosphoribosyl pyrophosphate (PRPP) that is the acceptor of adenine and hypoxanthine in the purine salvage pathway to form AMP and inosine monophosphate (IMP), in the reactions catalyzed by the enzymes adenine phosphoribosyl transferase (APRT) and hypoxanthine-guanine phosphoribosyl transferase (HGPRT), respectively. The IMP derived from hypoxanthine can be amidated to yield also AMP by the successive reactions of adenylosuccinate synthetase and adenylosuccinate lyase (Fig. 10).

The potential of brain cells for *de novo* synthesis of AMP is considered as rather low [235, 236], suggesting that synthesis of new AMP for subsequent ATP restoration depends mainly on the purine salvage pathway in astrocytes. All enzymes required for the salvage pathway are expressed in astrocytes [236], suggesting that these cells are able to use the purine bases adenine and hypoxanthine as well as their respective nucleosides, adenosine and inosine, as substrate for the synthesis of new AMP that will foster ATP restoration. Indeed, rapid and almost complete ATP restoration was found for ATP-depleted astrocytes 60 min after the application of glucose as energy substrate and of adenosine as substrate for AMP synthesis [60]. Under such conditions already micromolar concentrations of adenosine facilitate efficient ATP restoration. The adenosine-dependent ATP restoration is fully prevented by inhibition of adenosine kinase [60], demonstrating that this enzyme is highly efficient to phosphorylate adenosine in astrocytes to AMP which is further phosphorylated by the consecutive reactions of adenylate kinase and ADP phosporylating enzymes. A short isoform of adenosine kinase is expressed in astrocytes that is localized primarily in the cytosol and is responsible for rapid phosphorylation of uptaken adenosine [38].

ATP restoration was also found in ATP-depleted astrocytes after application of adenosine as exclusive substrate. However, in the absence of glucose higher adenosine concentrations are required to enable ATP restoration as under such conditions adenosine serves both as building block for AMP synthesis and as energy substrate to facilitate ATP restoration [60]. Accordingly, inhibition of adenosine kinase and inhibition of purine nucleoside phosphorylase lowers ATP restoration in astrocytes that had been fed with adenosine as exclusive substrate [60].

Astrocytes have been reported to convert adenosine via inosine to hypoxanthine [240], demonstrating that adenosine deaminase and purine nucleoside phosphorylase are active in these cells. As also the enzymes HGPRT (converts hypoxanthine to IMP) and adenylosuccinate synthase and adenylosuccinate lysase (amidate IMP to AMP) are expressed in astrocytes [236], it can be assumed that also inosine can serve as extracellular precursor for AMP synthesis and subsequently for ATP restoration. Indeed, partial ATP restoration was found after application of inosine plus glucose to ATP-depleted astrocytes [241]. Some ATP restoration also takes place after application of guanosine and glucose [241]. However, rapid and almost complete ATP restoration was reported for glucose-fed astrocytes after co-application of micromolar concentrations of adenine with inosine or guanosine in a process that was prevented by the presence of the purine nucleoside phosphorylase (PNP) inhibitor forodesine, suggesting an insufficient supply of PRPP from astrocytic glucose metabolism [241]. This limitation can be bypassed by the application of inosine or guanosine as source for additional ribose phosphate that accelerates the synthesis of the PRPP needed for the APRT-catalyzed formation of AMP.

ATP restoration experiments revealed that synthesis of new AMP is essential for the rapid restoration of normal ATP levels in ATP-depleted astrocytes and that this AMP synthesis via the purine salvage pathway requires an adenine precursor, a substrate that is efficiently metabolized to provide the energy for efficient ATP regeneration from ADP [60] as well as a suitable substrate that provides the ribose phosphate needed for AMP synthesis [241]. Further studies are now required to elucidate which combinations of energy substrates and AMP precursors will be best to facilitate rapid restoration of ATP in astrocytes.

## Future Perspectives

ATP depletion in brain has been reported for ischemic conditions and stroke [242–245] and for traumatic head injuries [235, 246, 247]. Although it remains to be elucidated to which extent a decline in astrocytic ATP contributes to the overall ATP loss in brain tissue under such conditions, hypoxic and ischemic conditions have been connected with a loss of ATP in astrocytes at least in cultures [68, 79] and in organotypic tissue slices [142].

So far, most of the studies that have addressed the ATP metabolism of astrocytes have been performed on cell cultures derived from murine brain or on brain slices from mouse brain. Recently, a first study reported data on monitoring alterations in ATP levels in astrocytes in the living brain [149]. Further studies are now required to elucidate the importance of a high ATP content in astrocytes in vivo and for maintaining the important functions of astrocytes for the living brain.

For restoration of ATP in ATP-deprived cultured rat astrocytes, the application of adenosine in presence of glucose was highly efficient as cellular adenosine is rapidly phosphorylated to AMP by adenosine kinase [60]. However, due to its strong neuroactive potential [59, 235, 248], an application of adenosine should not be considered for treatments to support ATP restoration in brain. Combinations of substrates that can provide the components of ATP (ribose, phosphate, adenine) and the energy for ATP restoration have been suggested to support ATP restoration in ATP-deprived brain cells [235]. Due to the potential of cultured astrocytes to use a broad range of substrates to fuel ATP regeneration and to maintain a high ATP content, cultured astrocytes may be a good model to screen for substance combinations that foster rapid ATP restoration via synthesis of new AMP and its subsequent phosphorylation to ATP. Combinations of an adenine-containing precursor, a substrate for the formation of ribose phosphate plus an energy substrate may be suitable to support efficient ATP restoration even in glucose-deprived astrocytes. Results of such studies may be helpful to design new strategies that could help to limit cerebral ischemia/reperfusion injury and to overcome the dilemma that glucose intervention failed to be benefical in stroke therapy [249].

ATP is consumed in several cellular compartments, but little is known on the concentrations of ATP present in these compartments and on the modulation of the respective ATP concentrations in astrocytes. The use of organelle-directing genetically encoded ATP sensors, for example mitochondria-targeted ATP sensors [250], may be useful to address such questions and to also investigate the trafficking of ATP and other adenosine phosphates between compartments. In this context, especially the mitochondrial adenine nucleotide translocase (ANT) has to be considered, a transporter of the mitochondrial inner membrane that exports mitochondrial ATP in antiport with cytosolic ADP [251]. Alterations in the expression or activity of the ANT have been connected with the activation of astrocytes [252, 253] and with hypertonicity [254]. Further studies are required to better understand the regulation of the expression and the activity of ANT and potential consequences of alterations in ANT activity on the exchange between the pools of adenosine phosphates in the cytosol and the mitochochondria, especially under conditions of impaired glycolysis and/or oxidative phosphorylation.

Astrocytes contain a high activity of CrK which represents mainly the cytosolic brain CrK isoform [173]. Also, in the human cortex strong immunoreactivity for CrK was found particularly in astrocytes [36], suggesting that CrP plays especially in astrocytes an important role as energy buffer. Interestingly, detectable immunoreactivity for CrK was low in astrocytes in the brains of patients with Alzheimer disease and evidence for different posttranslational modification of astrocytic CrK in Alzheimer disease was presented [36]. Further studies are required to elucidate in more detail the regulation of CrK in astrocytes, the intracellular distribution of CrK, the function of CrP as energy buffer for astrocytic ATP-consuming reactions and potential consequences of an altered astrocytic CrP metabolism in pathological conditions. Considering that the cellular levels of CrP can be increased in astrocytes by application of creatine [55], it should be studied whether creatine supplementation may also increase the level of CrP in astrocytes in the brain and whether such a process may contribute to the reported beneficial consequences of a creatine supplementation for the brain [133, 255, 256].

Astrocytes have a high potential to keep the cellular ATP content high and they make use of a broad range of endogenous and exogeneous substrates to fuel ATP regeneration. While the glycolytic glucose metabolism and the mitochondrial oxidation of glycolysis-derived pyruvate and amino acid-derived α-keto acids have been extensively studied for astrocytes, more information is warranted to understand the contribution of LDs [189, 190] and proline [70, 257] in the energy metabolism of astrocytes, especially considering the known consequences of alterations in the metabolism of LDs [190, 258] and proline [259] in human neurological diseases.

Little is known also on the ATP metabolism of human astrocytes. A recent study reported data on the ATP regeneration of human iPSC-derived astrocytes [122]. Such model systems for human brain cells may be suitable to study the pathways involved in the ATP restoration in ATP-deprived brain cells, which may help to develop new strategies to restore ATP contents in human brain after stroke [242–245] or traumatic head injuries [235, 246, 247].

## Data Availability

Enquieries on original data should be directed to the corresponding author.
